# Integrated whole genome and transcriptome analysis identified a therapeutic minor histocompatibility antigen encoded by an alternative ITGB2 transcript

**DOI:** 10.1186/2051-1426-3-S2-P285

**Published:** 2015-11-04

**Authors:** Margot J Pont, Dyantha van der Lee, Edith van der Meijden, Cornelis van Bergen, Michel Kester, Maria Honders, Martijn Vermaat, Matthias Eefting, Erik Marijt, Szymon Kielbasa, Peter-Bram 't Hoen, Frederik Falkenburg, Marieke Griffioen

**Affiliations:** 1 Department of Hematology, Leiden University Medical Center, Leiden, Netherlands; 2 Department of Human Genetics, Leiden University Medical Center, Leiden, Netherlands; 3 Department of Medical Statistics and Bioinformatics, Leiden University Medical Center, Leiden, Netherlands

## 

Patients with hematological malignancies can be successfully treated with allogeneic stem cell transplantation (alloSCT). In HLA-matched alloSCT, donor T cells can mediate desired anti-tumor immunity as well as undesired side effects by recognizing minor histocompatibility antigens (MiHA) on patient cells. MiHA are polymorphic peptides with amino acid changes that are created by genetic variants and recognized by specific T cells. MiHA with hematopoiesis restricted expression are relevant targets for immunotherapy to augment anti-tumor immunity after alloSCT without side effects.

For high-throughput discovery of MiHA, we previously developed whole genome association scanning (WGAs) in which T cell recognition of a panel of EBV-B cell lines is investigated for association with single nucleotide polymorphisms (SNPs) to identify the genomic region that encodes the antigen. Although WGAs is an efficient strategy for MiHA discovery, strong association with SNPs in introns or other regions outside coding exons can be found for approximately one third of the T cell clones, whereas no SNP disparity in the primary transcript is present between patient and donor, suggesting that the antigen may be encoded by an alternative transcript. To identify MiHA encoded by alternative transcripts, we developed an integrated approach in which WGAs is combined with whole transcriptome data from the GEUVADIS project.

By performing WGAs, we identified associating SNPs for two HLA-B*15:01-restricted MiHA that were targeted by donor CD8 T cells in a patient with strong anti-tumor immunity after HLA-matched alloSCT. One antigen (LB-GLE1-1V) was encoded by an associating SNP in exon 6 of the *GLE1* gene. For the other antigen, an associating SNP in intron 3 of the *ITGB2* gene was found, but no SNP disparity was present in the normal *ITGB2* transcript between patient and donor. Therefore, we investigated whether the antigen may be encoded by an alternative transcript. Using RNA-sequence data, we identified an alternative *ITGB2* transcript in which part of intron 3 was retained. Q-PCR analysis showed that expression of this transcript was restricted to hematopoietic cells from SNP-positive individuals. *In silico* translation of the transcript revealed a peptide with strong predicted binding to HLA-B*15:01 (LB-ITGB2-1), which was recognized by specific T cells. T cells for LB-ITGB2-1 also recognized and lysed leukemic cells of different origins, while no reactivity against patient fibroblasts could be detected.

In summary, RNA-sequence analysis enabled discovery of LB-ITGB2-1 as immune epitope encoded by an alternative gene transcript. Our data support the therapeutic relevance of LB-ITGB2-1 as target for T cell therapy to stimulate anti-tumor immunity after alloSCT.

